# The Bipolar Disorder Medication Adherence Battery: validity, reliability, and clinical benchmarks

**DOI:** 10.3389/fpsyt.2025.1731246

**Published:** 2026-02-19

**Authors:** Buket Ünver, Özlem Sertel Berk, Nesrin Karamustafalıoğlu

**Affiliations:** 1 Department of Psychiatry, Bakirköy Prof Mazhar Osman Training and Research Hospital for Psychiatry, Neurology, and Neurosurgery, Istanbul, Türkiye; 2 Department of Psychology, Istanbul University, Istanbul, Türkiye

**Keywords:** adherence, bipolar disorder, compliance, evidence-based assessment, scale validation

## Abstract

**Background:**

Medication adherence is pivotal in bipolar disorder, yet no tools assess the full range of psychological and contextual determinants that translate intention into behavior. This study developed and validated the Bipolar Disorder Medication Adherence Battery (BD-MAB) to support routine, measurement-based care.

**Methods:**

The analytic sample comprised 167 adults with DSM-5 bipolar I disorder recruited from outpatient and inpatient services at a tertiary psychiatric hospital. Guided by the Integrated Behavioral Model, we mapped process measures—Attitude; Perceived norms (supportive, oppositional, pressuring, descriptive); Perceived control; Self-efficacy; Knowledge and skills; Environmental Constraints; Salience of Behavior; Habit; Intention and a behavior indicator. Psychometrics included internal consistency; construct validity via inter-construct correlations; convergent and discriminant validity with the Medication Adherence Rating Scale, Medication Adherence Report Scale, perceived social support, and quality of life; criterion validity against same-day serum medication levels; and distribution-based thresholds (standard error of measurement, critical change, minimally important difference, reliable change index).

**Results:**

Administered to euthymic adults with bipolar I disorder receiving mood stabilizers at a tertiary psychiatric hospital, the BD-MAB showed acceptable to excellent internal consistency across multi-item scales. Relations among constructs were theory-consistent, with intention aligning most closely with self-efficacy and attitudes, and the behavior indicator tracking intention. Concordance with serum levels supported criterion validity, and associations with an established adherence measure supported convergent validity. Clinically interpretable thresholds were derived to flag meaningful change for use in routine care.

**Conclusions:**

The BD-MAB offers a comprehensive, clinically actionable assessment of adherence determinants and behavior in bipolar disorder, with initial evidence for reliability, validity, and practical change metrics to guide patient-level monitoring.

## 1 Introduction

Bipolar disorder (BD) is a chronic mood disorder marked by recurrent episodes of depression and (hypo)mania or mixed states, producing substantial emotional burden, behavioral dysregulation, and functional impairment (1).The onset typically occurs in late adolescence, peaking around age 20, with approximately one-third of cases beginning before age 25 (2). The lifetime prevalence of BD is estimated to range 2–4% (3, 4), with an increasing incidence over the past decades (5). After the onset of BD, many individuals experience cognitive, psychosocial, and occupational impairments even during euthymic phases (6, 7) and frequently develop chronic medical conditions (8, 9), burdening health care costs worldwide (10). Not surprisingly, BD ranks among the top 20 contributors to global disability (11, 12) and affected individuals attempt suicide up to 30 times more frequently than the general population (13, 14).

Pharmacotherapy is crucial for acute and maintenance treatment (15, 16). However, medication alone often does not achieve full symptomatic remission, functional recovery, or durable relapse prevention because of residual symptoms, subthreshold mood fluctuations, adherence challenges, and psychosocial stressors. Adjunctive psychosocial and psychotherapeutic interventions, when added to pharmacotherapy, have demonstrated superiority to medication alone in lowering recurrence risk, improving functioning, and enhancing adherence (17, 18). They are therefore considered core components of best practice in BD care (15, 19).

An evidence-based assessment (EBA) framework complements these treatments by structuring reliable, valid, and clinically useful assessment across the continuum of care. EBA uses measures at prediction (pre-visit screening/triage), prescription (intake diagnosis and case formulation), and process & progress (process and outcome, including clinician-patient relationship dynamics and ongoing symptoms) to improve case conceptualization, treatment selection, and measurement-based care (20, 21). The EBA approach integrates research evidence, clinical expertise, and patients’ experiences in order to enable timely adjustments of treatment (22).

Due to the clinical complexity, heterogeneity, medical and psychosocial comorbidity, and high-risk outcomes of the bipolar disorder, there is a need for a comprehensive, bipolar-specific EBA battery that assesses the full range of psychological and behavioral mechanisms underlying medication adherence. Importantly, medication adherence in bipolar disorder is shaped by state-dependent clinical dynamics; fluctuations in mood symptoms and variability in insight are closely linked in BD, and estimates of poor insight vary depending on the assessment method (23–25). Medication adherence—and the extent to which adherence intentions translate into actual medication-taking—may vary over the course of bipolar disorder, underscoring the need for a framework that captures multiple determinants of adherence (26–28). In BD, the episodic course with periods of symptomatic improvement can increase intentional discontinuation (“I am well now”), whereas manic/hypomanic states may further disrupt judgment and insight—making adherence determinants more phase-sensitive than in many chronic psychiatric conditions and warranting BD-specific assessment content (26, 27). Although the Integrated Behavior Model (IBM) is transdiagnostic, applying it to bipolar disorder is particularly informative given BD’s episodic course and phase-linked shifts in insight and motivation, while adherence assessment practices often remain largely shared across serious mental illnesses (29, 30). Existing tools often focus narrowly on self-reported adherence or isolated belief domains, overlooking the multiple determinants that shape adherence intention and its translation into actual behavior (30–33)(For example, medication adherence in bipolar disorder is most commonly assessed using brief self-report instruments that index either medication-taking behavior (e.g., MMAS-8, MARS-5, AMSQ) or medication-related beliefs/attitudes (e.g., DAI-10, BMQ-S), sometimes complemented by clinician ratings and objective indicators such as electronic monitoring (MEMS/EAM), pharmacy-refill metrics (e.g., PDC), pill counts, and therapeutic drug monitoring (TDM) (30, 33–35). In parallel, adherence tools are frequently used transdiagnostically across serious mental illnesses, despite clinically meaningful diagnostic and course-related differences—supporting the need for bipolar-disorder–specific assessment content grounded in a coherent, mechanism-focused framework (36, 37). This article introduces a new battery—the Bipolar Disorder Medication Adherence Battery (BD-MAB)—that captures both the determinants of intention and the direct behavioral enablers, offering a theoretically integrated and clinically useful framework for assessing and monitoring medication adherence and persistence in individuals with BD.

### 1.1 Brief description of the development of the Bipolar Disorder Medication Adherence Battery (BD-MAB)

Grounded in the integrated behavioral model (IBM), the BD-MAB was developed to address gaps in mechanism-focused assessment and monitoring for routine bipolar care (29). The IBM posits that intention is the most proximal predictor of behavior; however, effective behavior change also depends on knowledge and skills, environmental constraints, salience and accessibility, and habitual tendencies (29). Accordingly, development began by mapping IBM constructs to routine medication use in bipolar disorder and conducting a comprehensive review of adherence and conceptually related assessment tools to identify content that could be adapted and gaps requiring new items; this work highlighted limitations of existing tools and justified building a brief yet theoretically complete battery.

Guided by construct definitions and measurement recommendations in the IBM literature (29) a 150-item pool was created by a research team composed of 3 psychiatrists, 2 clinical psychologists, and 5 individuals with lived experience of BD, who provided brief interview-based feedback to refine item relevance and wording, to cover attitude, subjective norms, perceived control, self-efficacy, knowledge, environmental constraints, salience, habit, intention, and behavior. To ensure the item pool reflected the model’s full scope, the model was operationalized to capture both intention formation and the translation of intention into behavior; this approach may provide a potentially informative framework in bipolar disorder, where the relative weight of adherence mechanisms may plausibly vary across clinical phases. Where suitable, items were adapted from established scales (e.g., beliefs about medicines, treatment attitudes, lithium/mood-stabilizer attitudes, practical barriers (38, 39); see **Supplementary Table S1** for the full list of source **instruments**); where no adequate measures existed for a construct, new items were written directly from IBM specifications (see **Supplementary Table S1**). This item pool produced a 13-measure battery reflecting the full IBM determinant set relevant to medication adherence.

The initial battery was piloted on a sample of outpatients with BD in remission (N = 48; predominantly BD-I; mean illness duration ≈ 11 years). Items were evaluated for distribution, item–total relations, and clarity within each construct. The final structure of the BD-MAB was established in the main validation study through principal component analysis, which reduced the initial 109 items to 91 by retaining those with optimal psychometric performance while preserving the theoretical structure (40).

The final version of the BD-MAB comprises 91 items reflecting the IBM process measures (i.e., attitude, subjective norms, perceived control, self-efficacy, knowledge, environmental constraints, salience, habit, and intention) plus a behavior indicator. Its psychometric properties are tested and reported in this article.

This stepwise development (literature review, item generation/adaptation, expert and language review, piloting with item analysis, and psychometric evaluation) follows best practices for psychiatric and psychological scale development (41). An expert panel (two academic clinicians and a senior psychiatrist) reviewed construct coverage and item relevance, guided the selection/mapping of candidate measures to IBM constructs, and informed additional item writing when needed; translated items underwent forward translation (two English-language specialists and a psychiatrist) and language/clarity review (two Turkish-language specialists and an independent psychiatrist), followed by piloting for comprehensibility.

### 1.2 Measures included in the BD-MAB

**Figure 1:**
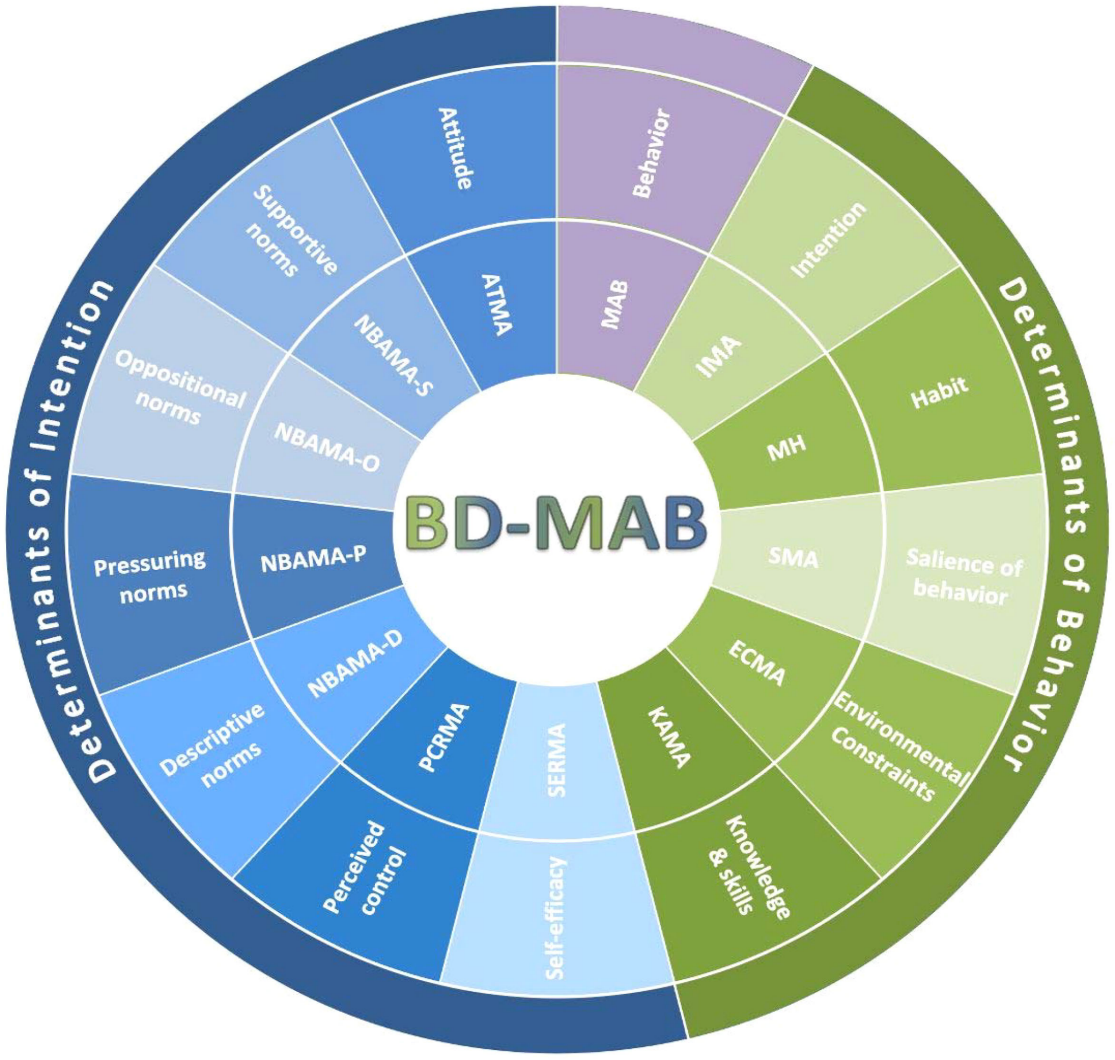
The structure of the Bipolar Disorder Medication Adherence Battery (BD-MAB) within the integrated behavioral model. The inner ring displays the acronyms for the BD-MAB scales; the middle ring shows their corresponding Integrated Behavioral Model (IBM) domains; and the outer ring groups these domains into the IBM macrodomains: determinants of intention (blue semicircle) and determinants of behavior (green semicircle). ATMA, Attitude Toward Medication Adherence; ECMA, Environmental Constraints to Medication Adherence; IMA, Intention of Medication Adherence; KAMA, Knowledge About Medication Adherence; MAB, Medication Adherence Behavior; MH, Medication Habit; NBAMA-D, Normative Beliefs about Medication Adherence—Descriptive Norms; NBAMA-O, Normative Beliefs about Medication Adherence—Oppositional Norms; NBAMA-P, Normative Beliefs about Medication Adherence—Pressuring Norms; NBAMA-S, Normative Beliefs about Medication Adherence—Supportive Norms; PCRMA, Perceived Control Regarding Medication Adherence; SERMA, Self-Efficacy Regarding Medication Adherence; SMA, Salience of Medication Adherence.

The BD-MAB comprises 13 self-report instruments organized according to the IBM (**Figure 1**). It includes scales representing the determinants of intention—attitude, perceived norms (supportive, oppositional, pressuring, descriptive), perceived control, and self-efficacy—as well as scales assessing the direct determinants of behavior: intention, knowledge and skills, salience, environmental constraints, and habit. Together, these components provide a comprehensive framework for explaining and monitoring medication adherence behavior in individuals with BD. Each scale is described below, with complete instruments and scoring instructions in the **Supplementary Appendix**.

The Attitude Toward Medication Adherence (ATMA) scale is a 26-item measure assessing individuals’ general attitudes toward psychiatric medication. Each item is rated on a 7-point Likert scale, with higher scores reflecting more positive attitudes.

The Normative Beliefs about Medication Adherence (NBAMA) is a 25-item set of four scales (no total score) assessing perceived social influences on recent medication adherence. The scales are: Supportive Norms (6 items), Oppositional Norms (6 items), Pressuring Norms (6 items), and Descriptive Norms (7 items). Each item is rated on a 7-point Likert scale. Scores are computed separately for each subscale, with higher scores indicating greater endorsement of the corresponding normative influence. A total score is not calculated because the four subscales capture conceptually distinct and potentially co-occurring normative pressures (e.g., supportive and pressuring influences may be experienced simultaneously from different sources); therefore, they are interpreted as separate indicators rather than as a single latent dimension.

The Perceived Control Regarding Medication Adherence (PCRMA) scale is a 9-item measure assessing individuals’ perceptions of how easy vs. difficult it is for them to control and manage factors related to taking medication as prescribed. Each item is rated on a 7-point Likert scale, with higher scores indicating greater perceived control over taking meds as prescribed.

The Self-Efficacy Regarding Medication Adherence (SERMA) scale is a 12-item measure assessing individuals’ confidence in their ability to adhere to medication despite perceived difficulties or barriers. In the IBM framework, self-efficacy refers to the extent to which a person feels confident in performing the behavior despite the perceived challenges or obstacles. Each item is rated on a 7-point Likert scale, with higher scores reflecting greater confidence to adhere despite challenges.

The Knowledge About Medication Adherence (KAMA) scale is a 7-item measure assessing individuals’ factual knowledge and skills about proper medication use. Each item is answered with response options of “True,” “False,” or “Unsure,” with higher total scores indicating more correct medication knowledge.

Environmental Constraints to Medication Adherence (ECMA) scale is a 5-item measure assessing commonly experienced barriers to consistent medication use. Each item is rated on a 7-point Likert scale; higher scores reflect greater perceived barriers.

Salience of Medication Adherence (SMA) is a 4-item measure assessing the respondent’s perceived ease of access to prescribed medications, including factors such as financial, social, and logistical support. Each item is rated on a 7-point Likert scale, with higher total scores indicating greater ease of access.

The Medication Habit (MH) scale is a single-item measure evaluating the extent to which taking medication has become a regular, automatic behavior, based on current habits. The item is rated on a 7-point Likert scale, with a higher score indicating a stronger and more regular habit.

The Intention of Medication Adherence (IMA) scale is a single-item measure that assesses the individual’s likelihood of adhering to prescribed medication over the next three months. The item is rated on a 7-point Likert scale, with a higher score indicating stronger intention to adhere.

The Medication Adherence Behavior (MAB) scale is a single-item measure evaluating the proportion of prescribed medication taken during the past week. The item is rated on a 5-point Likert scale ranging from 0% (none) to 100% (all).

## 2 Methods

### 2.1 Participants

The analytic sample consisted of 167 adults (M_age_ = 39.8 years, SD = 12) with a DSM-5. diagnosis of bipolar I disorder. 3.6% (*n* = 6) had a comorbid Axis II (personality) disorder. The sample comprised 64% women and 36% men (*n* = 60). Most participants had either a primary (40%, *n* = 67) or high school (24%, *n* = 40) education. Regarding marital status, 55% were married, 35% single, and 10% divorced. The majority (92%, *n* = 153) were assessed in outpatient settings, and the duration of illness (i.e., time since official diagnosis) was 11.7 years (SD = 9.4; range = 1–40) on average. A history of suicide attempt was reported by 25% of participants (*n* = 41), while 75% (*n* = 126) denied prior attempts. Lifetime suicidal ideation was endorsed by 44% (*n* = 74). The average number of lifetime hospitalizations was 3.4 (SD = 4.2; range = 0–24). At study entry, participants were prescribed mood stabilizers: lithium (49%, *n* = 82), valproate (48%, *n* = 80), and carbamazepine (2%, *n* = 4). Serum levels were obtained clinically on the same day and used to assess objective medication adherence. Antipsychotic exposure, measured in chlorpromazine-equivalent daily doses, did not significantly differ between adherence groups (adherent: M = 318.3, SD = 280.7; non-adherent: M = 384.3, SD = 268.3), *t*_(141)_ = −0.959, *p* >.05. The average number of psychotropic medications per participant was 5.5 (SD = 2.5; range = 1–19).

### 2.2 Procedures

Participants were recruited at the Bakırköy Mazhar Osman Mental Health and Neurological Diseases Education and Research Hospital (Istanbul, Turkey) and assessed in either outpatientclinics or inpatient wards. Recruitment was coordinated with treating psychiatrists.

Inclusion criteria were: (a) a DSM-5 diagnosis of bipolar disorder, (b) at least primary school education, (c) age between 18 and 75 years, (d) a Hamilton Depression Rating Scale score of ≤7 at inclusion (42) indicating absence of depressive symptoms, (e) a Young Mania Rating Scale score of ≤5 (43), indicating absence of manic symptoms, and (f) regular use of a mood stabilizer for at least one month. Exclusion criteria was having a diagnosis of substance use disorder.

Blood sampling for routine serum mood-stabilizer levels occurred as part of standard clinical care prior to study assessment; laboratory results served as the objective adherence indicator. Assessments followed a standardized sequence in a quiet room. The BD-MAB components were administered via individual interviews, while other measures were self-completed with the researcher present but non-intervening; assistance was provided when necessary. Across the recruitment period, 190 patients were approached and assessed. After excluding incomplete or erroneously completed forms, the final analytic sample comprised 167 complete cases.

The study protocol was approved by the Bakirköy Psychiatric Hospital Clinical Research Ethics Committee (No. 2017/94). Written informed consent was obtained after participants received oral and written information about the study. Participation was voluntary, confidentiality was assured, and procedures adhered to institutional and international ethical standards, including the Declaration of Helsinki.

### 2.3 Measures

Alongside the BD-MAB, we administered the following measures to examine criterion-related, convergent, and discriminant validity.

A sociodemographic and clinical data form was used to collect self-reported information about age, sex, education, marital status, and current psychopharmacological treatment. Treating clinicians recorded the primary diagnosis and any substance use disorders.

The Hamilton Depression Rating Scale (HDRS (42, 44); is a 21-item clinician-administered scale that assesses the severity of depressive symptoms over the past week. Each item is rated on a 5-point Likert scale, with higher scores indicating more severe depressive symptoms.

The Young Mania Rating Scale (YMRS (43, 45); is an 11-item clinician-administered scale used to evaluate the severity of manic symptoms experienced during the previous week. Each item is rated on a 5-point scale, where higher scores denote greater symptom severity.

The Multidimensional Scale of Perceived Social Support (MSPSS (46, 47); is a 12-item self-report questionnaire designed to assess perceived social support from three sources: Family, Friends, and Significant Other. Each item is rated on a 7-point Likert scale, with higher scores reflecting greater perceived support.

The WHO Quality of Life Scale–BREF (WHOQOL-BREF-TR (48); is a 27-item self-report instrument that measures quality of life across four domains: Physical Health, Psychological Well-being, Social Relationships, and Environmental Context. Each item is rated on a 5-point Likert scale, with higher scores indicating better quality of life.

The Medication Adherence Rating Scale (MARS (49, 50); is a 10-item self-report scale assessing medication adherence over the past week. Each item is answered with a binary “yes” or “no” response, with higher total scores indicating better adherence.

The Medication Adherence Report Scale (MARS-5 (51, 52); is a 5-item self-report scale designed to assess adherence to medication regimens. Each item is rated on a 5-point Likert scale. Higher total scores indicate greater adherence.

Serum levels of mood stabilizers (lithium, valproic acid, or carbamazepine) were obtained on the same day as the self-report measures and served as an objective biomarker of the adherence-behavior construct. Levels within the therapeutic range (lithium: 0.50–1.00 mEq/L; valproic acid: 50–100 mEq/L; carbamazepine: 6–12 mEq/L) were coded as “adherent,” while levels outside this range were coded as “non-adherent.”

### 2.4 Statistical analysis

Participants’ demographic and clinical characteristics and scale scores were summarized with descriptive statistics (frequencies and percentages). Criterion-related, convergent, and discriminant validity, were evaluated using Pearson product–moment correlations (*r*). Normality was evaluated using skewness and kurtosis indices and Q–Q plots (53). Most variables met commonly used normality cutoffs, whereas a small subset showed departures from normality, primarily reflected in elevated kurtosis; accordingly, Pearson correlations were estimated and percentile bootstrap 95% confidence intervals were computed for all correlations (5,000 resamples). Correlation strengths are interpreted as follows: very weak (0.00–0.19), weak (0.20–0.39), moderate (0.40–0.59), strong (0.60–0.79), and very strong (0.80–1.00) (54). Internal consistency was assessed with Cronbach’s α; values ≥.70 were considered adequate, ≥.80 good, and ≥.90 excellent (20). For brief scales assessing broad constructs, lower α (≈.50) may be acceptable (55). For short measures, the average interitem correlation (AIIC) is often more informative than α (56), with a recommended range of .15–.50 to balance desirable commonality and avoid redundancy among items (57). Internal consistency cannot be computed for single-item scales. Lastly, to enhance clinical utility of the battery, for each scale the standard error of measurement (SE_m_) and the standard error of the difference (SE_d_) were calculated to quantify score variability attributable to measurement error; critical change values at the 90% and 95% confidence levels to indicate the minimum change exceeding error; the minimally important difference (0.5 × SD) was used as a benchmark for clinically meaningful change; and the minimum change for a reliable change (58) was applied to classify statistically reliable improvement or deterioration. To test the hypothesized IBM pathways, we additionally conducted a structural equation modeling (SEM) analysis at the construct-score level. Intention was regressed on attitude, perceived norms (supportive, oppositional, pressuring, descriptive), perceived control, and self-efficacy; behavior was regressed on intention, habit, salience (access), environmental constraints, and knowledge. The SEM was estimated using maximum likelihood with robust standard errors, and model fit was evaluated using the Yuan–Bentler T2* scaled χ², SRMR, and robust RMSEA/CFI/TLI. SEM analyses were conducted in jamovi (SEMLj; lavaan), using listwise deletion for missing data (N = 167). All other analyses were conducted in SPSS Version 22.0.

## 3 Results

### 3.1 Descriptive statistics and internal consistency

Scale means, SDs, AIICs, and Cronbach’s α coefficients are reported in **Table 1**. AIICs ranged from .16 to.53, indicating adequate to excellent internal consistency across the BD-MAB scales.

**Table 1:** Descriptive, reliability, and change metrics for BD-MAB scales.

Scale	IBM domain	*k*	M (SD)	α	AIIC	SE_m_	SE_d_	CC_90_	CC_95_	MID	RCI
ATMA	Attitude	26	138.40 (21.63)	.90	.27	6.84	9.67	11.3	13.4	10.8	19.0
NBAMA-S	Supportive norms	6	34.49 (6.04)	.67	.23	3.47	4.91	5.7	6.8	3.0	9.6
NBAMA-O	Oppositional norms	6	36.66 (5.89)	.58	.18	3.82	5.40	6.3	7.5	2.9	10.6
NBAMA-P	Pressuring norms	6	36.57 (7.49)	.73	.22	3.89	5.50	6.4	7.6	3.8	10.8
NBAMA-D	Descriptive norms	7	34.64 (7.27)	.77	.24	3.49	4.93	5.7	6.8	3.6	9.7
PCRMA	Perceived control	9	47.12 (9.83)	.84	.19	3.93	5.56	6.5	7.7	4.9	10.9
SERMA	Self-efficacy	12	71.66 (9.57)	.92	.53	2.71	3.83	4.5	5.3	4.8	7.5
KAMA	Knowledge & skills	7	12.95 (1.22)	.58	.35	0.79	1.12	1.3	1.6	0.6	2.2
ECMA	Environmental constraints	5	30.25 (4.24)	.69	.39	2.36	3.34	3.9	4.6	2.1	6.5
SMA	Salience of behavior	4	26.60 (2.41)	.45	.16	1.79	2.53	2.9	3.5	1.2	5.0
MH	Habit	1	5.51 (1.37)	—	—	—	—	—	—	—	—
IMA	Intention	1	6.43 (0.99)	—	—	—	—	—	—	—	—
MAB	Behavior	1	4.42 (1.10)	—	—	—	—	—	—	—	—

α, Cronbach’s alpha; AIIC, average interitem correlation; ATMA, Attitude Toward Medication Adherence; CC_90/95_, critical change at the 90% or 95% confidence level (minimum score change to be 90/95% certain that true change has occurred); ECMA, Environmental Constraints to Medication Adherence; IBM, Integrated Behavioral Model; IIC, Inter-Item Correlations; IMA, Intention to Adhere to Medication; KAMA, Knowledge About Medication Adherence; *k*, number of items; MAB, Medication Adherence Behavior; MH, Medication Habit; MID, 0.5 × SD rule-of-thumb for the smallest clinically important difference; NBAMA, Normative Beliefs About Medication Adherence; NBAMA-D, Normative Beliefs about Medication Adherence—Descriptive Norms; NBAMA-O, Normative Beliefs about Medication Adherence—Oppositional Norms; NBAMA-P, Normative Beliefs about Medication Adherence—Pressuring Norms; NBAMA-S, Normative Beliefs about Medication Adherence—Supportive Norms; PCRMA, Perceived Control Regarding Medication Adherence; RCI, Jacobson–Truax reliable-change index; SE_d_, standard error of the difference for two administrations in the same phase; SE_m_, standard error of measurement (one-occasion error); SERMA, Self-Efficacy Regarding Medication Adherence; SMA, Salience of Medication Adherence.

### 3.2 Construct validity

Inter-construct correlations within the BD-MAB supported the IBM structure. Intention correlated strongly with self-efficacy (*r* = .75, very strong) and attitude (*r* = .62, strong), and the adherence behavior item correlated very strongly with intention (*r* = .76) and strongly with self-efficacy (*r* = .68) and attitude (*r* = .56). Oppositional and pressuring subjective norms showed small negative associations with intention (*r* = −.18 and −.14, respectively). Salience showed near-zero links with most constructs, consistent with its separable role (e.g., *r* = .07 with intention; *r* = −.01 with behavior). Overall, inter-construct significant *r*s ranged |.15–.76|. Complete inter-construct correlations among BD-MAB scales are reported in **Table 2**.

**Table 2:** Intercorrelations among BD-MAB scales.

	Scale	IBM domain	1	2	3	4	5	6	7	8	9	10	11	12	13
1	ATMA	Attitude	1												
2	NBAMA-S	Supportive norms	.30^**^ [.13,.46]	1											
3	NBAMA-O	Oppositional norms	-.18^*^ [-.32, –.03]	-.14 [-.29,.01]	1										
4	NBAMA-P	Pressuring norms	–.25^**^ [-.40, –.03]	.13 [-.04,.23]	.28^**^ [.15,.46]	1									
5	NBAMA-D	Descriptive norms	.34^**^ [.21,.47]	.47^**^ [.34,.58]	-.08 [-.23,.06]	.03 [-.11,.17]	1								
6	PCRMA	Perceived control	.50^**^ [.37,.61]	.19^*^ [.01,.37]	-.08 [-.23,.08]	-.04 [–.20,.09]	.35^**^ [.16,.52]	1							
7	SERMA	Self-efficacy	.63^**^ [.49,.73]	.30^**^ [.10,.49]	-.04 [-.20,.11]	-.07 [-.25,.08]	.23^**^ [.07,.38]	.69^**^ [.61,.76]	1						
8	KAMA	Knowledge & skills	.25^**^ [.07,.42]	.20^**^ [.05,.35]	–.00 [-.13,.13]	.00 [-.14,.12]	.11 [-.03,.26]	.34^**^ [.22,.47]	.44^**^ [.30,.56]	1					
9	ECMA	Environmental Constraints	.44^**^ [.27,.59]	.21^**^ [.04,.37]	-.09 [-.25,.05]	-.32^**^ [-.48. -.15]	.18^*^ [-.03,.37]	.43^**^ [.24,.59]	.46^**^ [.33,.58]	.22^**^ [.07,.39]	1				
10	SMAS	Salience of Behavior	-.01 [-.13,.13]	.12 [-.04,.30]	-.15 [-.31, -.01]	-.07 [-.22,.05]	.00 [-.17,.17]	.01 [-.13,.14]	.08 [-.05,.21]	.15 [.01,.29]	.18^*^ [.01,.38]	1			
11	MH	Habit	.41^**^ [.25,.55]	.26^**^ [.05,.35]	-.06 [-.23,.11]	-.00 [-.13,.11]	.25^**^ [.11,.40]	.42^**^ [.27,.55]	.56^**^ [.42,.68]	.31^**^ [.14,.46]	.29^**^ [.11,.47]	.03 [-.10,.16]	.1		
12	TAM	Intention	.62^**^ [.49,.72]	.30^**^ [.12,.47]	-.13 [-.32,.06]	-.16^*^ [-.34, -.01]	.22^**^ [.09,.35]	.44^**^ [.32,.56]	.75^**^ [.65,.83]	.42^**^ [.24,.57]	.36^**^ [.20,.52]	.07 [-.05,.21]	.56^**^ [.41,.69]	1	
13	MAB	Behavior	.56^**^ [.38,.68]	.24^**^ [.06,.41]	-07 [-.26,.11]	-.08 [-.27,.09]	.16^*^ [.02,.31]	.39^**^ [.26,.52]	.68^**^ [.54,.76]	.26^**^ [.07,.44]	.33^**^ [.15,.48]	–.01 [.11,.13]	.52^**^ [.34,.67]	.76^**^ [.64,.83]	1

Values are Pearson correlations. Bracketed values indicate 95% bootstrap confidence intervals (percentile method; 5,000 resamples). Scale ATMA, Attitude Toward Medication Adherence; ECMA, Environmental Constraints to Medication Adherence; IBM, Integrated Behavioral Model; KAMA, Knowledge About Medication Adherence; MAB, Medication Adherence Behavior; MH, Medication Habit; NBAMA, Normative Beliefs About Medication Adherence; NBAMA-D, Normative Beliefs about Medication Adherence—Descriptive Norms; NBAMA-O, Normative Beliefs about Medication Adherence—Oppositional Norms; NBAMA-P, Normative Beliefs about Medication Adherence—Pressuring Norms; NBAMA-S, Normative Beliefs about Medication Adherence—Supportive Norms; PCRMA, Perceived Control Regarding Medication Adherence; SERMA, Self-Efficacy Regarding Medication Adherence; SMAS, Salience of Medication Adherence; TAM, Intention to Adhere to Medication.

^**^p < 0.01, ^*^p < 0.05.

In addition to correlational evidence, the IBM structural model was tested using structural equation modeling (SEM). Intention was regressed on attitude, perceived norms (supportive, oppositional, pressuring, descriptive), perceived control, and self-efficacy; behavior was regressed on intention, habit, salience/access, environmental constraints, and knowledge. Model parameters were estimated using maximum likelihood (ML) with robust standard errors, and Yuan–Bentler T2* scaled fit statistics were reported (N = 167). Model fit was acceptable: χ²_YB(T2*)(11) = 21.20, p = .031 (χ²(11) = 26.60, p = .005); SRMR = .022; RMSEA_robust = .083 (95% CI [.024, .137]; RMSEA = .092, 95% CI [.048, .137]); CFI_robust = .960 (CFI = .952) and TLI_robust = .916 (TLI = .899). The model explained 63.0% of the variance in NIYT (R² = .630) and 59.5% of the variance in DAVR (R² = .595). Regarding path coefficients, at the intention level the positive effect of attitude was significant (attitude → intention: β = .227, z = 2.709, p = .007), and perceived control was negatively associated with intention (perceived control → intention: β = −.183, z = −2.640, p = .008). Self-efficacy was the strongest predictor of intention (self-efficacy → intention: β = .713, z = 6.976, p <.001). Direct effects of supportive norms (supportive norms → intention: β = .042, p = .464), oppositional norms (oppositional norms → intention: β = −.056, p = .398), pressuring norms (pressuring norms → intention: β = −.049, p = .362), and descriptive norms (descriptive norms → intention: β = .024, p = .687) were not significant. Although a high correlation was observed between intention and self-efficacy (r = .753), collinearity diagnostics for the intention equation were not problematic (tolerance = .389–.880; VIF = 1.136–2.569). At the behavior level, intention was the strongest predictor of behavior (intention → behavior: β = .696, z = 13.892, p <.001), and habit provided an additional contribution (habit → behavior: β = .142, z = 2.083, p = .037). Direct effects of salience/access (salience/access → behavior: β = −.056, p = .201), environmental constraints (environmental constraints → behavior: β = .063, p = .286), and knowledge (knowledge → behavior: β = −.077, p = .143) did not reach statistical significance.

### 3.3 Convergent and discriminant validity

Correlations with external validators were patterned and theory-consistent. Social support (MSPSS) and quality of life (WHOQOL-BREF) subscales were largely unrelated to subjective-norm pressure, barriers, and access (e.g., access with total social support *r* = .01; with QoL total *r* = .14), but showed small-to-moderate positive links with theoretically proximal determinants such as attitudes, perceived control, self-efficacy, habit, and intention (e.g., QoL total with attitudes *r* = .45; with self-efficacy *r* = .43; with intention *r* = .40). Medication adherence scales also converged as expected: MARS correlated strongly with BD-MAB intention (*r* = .65), habit (*r* = .47), and self-efficacy (*r* = .60); MARS-5 showed moderate correlations with habit (*r* = .57), self-efficacy (*r* = .56), perceived control (*r* = .58), and attitudes (*r* = .47). These patterns indicate convergent relations for core IBM determinants and discriminant relations for norm-pressure, barriers, and access. Complete correlations with external validators are presented in **Table 3**.

**Table 3:** Correlations between BD-MAB scales and external measures.

		Bipolar Disorder Medication Adherence Battery (BD-MAB)											
External Measure	M (SD)	ATMA	NBAMA-S	NBAMA-O	NBAMA-P	NBAMA-D	PCRMA	SERMA	KAMA	ECMA	SMAS	MH	TAM
BbMSPSS													
Family	22.62 (4.21)	.27^**^ [.09,.43]	.30^**^ [.15,.43]	-.00 [-.17,.16]	.15 [.03,.26]	.19^*^ [.03,.35]	.21^**^ [.04,.36]	.25^**^ [.03,.44]	.17^*^ [-.01,.34]	-.01 [-.18,.17]	.01 [-.17,.20]	.31^**^ [.12,.47]	.25^**^ [.01,.46]
Significant Other	14.92 (8.12)	.23^**^ [.10,.36]	.25^**^ [.10,.39]	.13 [-.02,.27]	.11 [-.07,.27]	.29^**^ [.14,.43]	.33^**^ [.18,.45]	.30^**^ [.17,.41]	.20^*^ [.06,.33]	.07 [-.10,.20]	-.04 [-.18,.09]	.30^**^ [.16,.42]	.20^*^ [.06,.31]
Friends	16.05 (6.73)	.18^*^ [.01,.33]	.33^**^ [.18,.48]	.09 [-.06,.23]	.10 [-.05,.24]	.31^**^ [.16,.45]	.26^**^ [.10,.40]	.25^**^ [.08,.39]	.24^**^ [.09,.38]	.11 [-.08,.26]	.05 [-.11,.20]	.20^**^ [.04,.35]	.21^**^ [.05,.34]
Total	53.59 (15.22)	.28^**^ [.13,.41]	.36^**^ [.21,.50]	.10 [-.05,.26]	.14 [-.01,.29]	.35^**^ [.20,.48]	.35^**^ [.20,.47]	.34^**^ [.20,.47]	.26^**^ [.11,.39]	.08 [-.03,.21]	.00 [-.16,.16]	.33^**^ [.18,.47]	.27^**^ [.12,.40]
WHOQOL-BREF-TR													
General health	6.68 (1.29)	.46^**^ [.31,.58]	.29^**^ [.14,.43]	-.10 [-.23,.04]	-.24^**^ [-.36, -.11]	.31^**^ [.18,.43]	.37^**^ [.23,.50]	.33^**^ [.18,.47]	.16^*^ [.00,.31]	.14 [-.06,.32]	.05 [-.10,.21]	.30^**^ [.15,.44]	.35^**^ [.21,.48]
Physical health	27.81 (3.41)	.38^**^ [.23,.51]	.18^*^ [.02,.35]	-.16^*^ [-.30, -.02]	-.27^**^ [-.44, -.09]	.16^*^ [.02,.31]	.35^**^ [.21,.47]	.32^**^ [.16,.47]	.20^**^ [.06,.34]	.13 [-.05,.29]	.09 [-.11,.30]	.30^**^ [.15,.44]	.32^**^ [.18,.45]
Psychological well-being	21.27 (3.02)	.40^**^ [.25,.53]	.39^**^ [.25,.52]	-.10 [-.25,.05]	-.13 [-.25, -.00]	.31^**^ [.16,.45]	.35^**^ [.21,.48]	.31^**^ [.17,.44]	.19^*^ [.03,.33]	.11 [-.05,.28]	.15^*^ [-.05,.36]	.32^**^ [.18,.44]	.30^**^ [.18,.42]
Social relationships	8.77 (2.33)	.23^**^ [.08,.37]	.39^**^ [.25,.52]	.06 [-.08,.20]	.01 [-.11,.14]	.32^**^ [.17,.46]	.33^**^ [.19,.45]	.27^**^ [.12,.41]	.31^**^ [.16,.43]	.11 [-.03,.25]	.07 [-.12,.24]	.25^**^ [.09,.39]	.28^**^ [.14,.40]
Environmental context	28.77 (3.83)	.31^**^ [.19,.43]	.29^**^ [.14,.43]	-.02 [-.17,.14]	.02 [-.13,.18]	.29^**^ [.14,.43]	.40^**^ [.28,.53]	.40^**^ [.27,.51]	.33^**^ [.19,.45]	.05 [-.10,.21]	.14 [.01,.28]	.43^**^ [.30,.54]	.32^**^ [.20,.42]
Total	93.30 (10.70)	.45^**^ [.32,.56]	.39^**^ [.25,.52]	-.08 [-.23,.07]	-.14 [-.25, -.03]	.35^**^ [.22,.48]	.47^**^ [.36,.57]	.43^**^ [.30,.54]	.32^**^ [.18,.44]	.13 [-.03,.29]	.15 [-.03,.33]	.43^**^ [.28,.55]	.40^**^ [.28,.50]
MARS	17.39 (2.09)	.68^**^ [.58,.76]	.19^*^ [.00,.36]	-.07 [-.24,.11]	-.17^*^ [-.36,.01]	.16^*^ [.03,.28]	.38^**^ [.26,.49]	.60^**^ [.45,.70]	.21^**^ [.02,.38]	.35^**^ [.19,.49]	.04 [-.09,.19]	.47^**^ [.32,.59]	.65^**^ [.51,.74]
MARS-5	20.62 (3.65)	.47^**^ [.30,.60]	.22^**^ [.03,.41]	.02 [-.13,.15]	-.16^*^ [-.31, -.02]	.29^**^ [.12,.44]	.58^**^ [.44,.69]	.56^**^ [.40,.68]	.34^**^ [.18,.49]	.44^**^ [.22,.59]	.15 [.00,.30]	.57^**^ [.41,.70]	.51^**^ [.33,.66]

Values are Pearson correlations. Bracketed values indicate 95% bootstrap confidence intervals (percentile method; 5,000 resamples). ATMA, Attitude Toward Medication Adherence; ECMA, Environmental Constraints to Medication Adherence; IBM, Integrated Behavioral Model; KAMA, Knowledge About Medication Adherence; MH, Medication Habit; NBAMA, Normative Beliefs About Medication Adherence; NBAMA-D, Normative Beliefs about Medication Adherence—Descriptive Norms; NBAMA-O, Normative Beliefs about Medication Adherence—Oppositional Norms; NBAMA-P, Normative Beliefs about Medication Adherence—Pressuring Norms; NBAMA-S, Normative Beliefs about Medication Adherence—Supportive Norms; PCRMA, Perceived Control Regarding Medication Adherence; SERMA, Self-Efficacy Regarding Medication Adherence; SMAS, Salience of Medication Adherence; TAM, Intention to Adhere to Medication. MARS, Medication Adherence Rating Scale; MARS-5, Medication Adherence Rating Scale (5-item version); MSPSS, Multidimensional Scale of Perceived Social Support; WHOQOL-BREF-TR, World Health Organization Quality of Life Scale. Values are Pearson correlations.^**^p < 0.01, ^*^p < 0.05.

### 3.4 Criterion-related validity

The BD-MAB adherence behavior item correlated strongly with MARS (*r* = .73) and moderately with MARS-5 (*r* = .50), and showed a strong association with serum drug level in the expected direction given the coding used (*r* = −.60), all *p* <.01. MARS and MARS-5 also correlated with serum levels (*r* = −.50 and *r* = −.36, respectively), supporting criterion convergence across methods.

Using a dichotomous adherence rule for the BD-MAB behavior item (scores 4–5 = adherent), cross-tabulation against serum-based adherence yielded: 130 adherent–adherent, 3 adherent on serum but non-adherent on BD-MAB, 18 non-adherent on serum but adherent on BD-MAB, and 16 non-adherent–non-adherent; χ² _(1)_ = 53.908, *p* <.001. From these counts: sensitivity = 97.7% (130/133), specificity = 47.1% (16/34), positive predictive value = 87.8% (130/148), negative predictive value = 84.2% (16/19), overall accuracy = 87.4% (146/167), and Matthews correlation coefficient (φ) ≈ .57 (large). These indices show excellent detection of adherent cases and moderate discrimination of non-adherence relative to the serum criterion.

### 3.5 Reliable and clinically meaningful change

To support patient monitoring, we report thresholds for critical change at 90% and 95% confidence (CC_90_ and CC_95_), minimal important difference (MID), and the reliable change index (RCI). For each BD-MAB scale score, compute the change Δ = follow-up − baseline and compare |Δ| with MID, CC_90/95_, and RCI. Interpret as follows:

- |Δ| < MID and |Δ| < CC_90_: no clinically meaningful or statistically reliable change.
- MID ≤ |Δ| < CC_90_: clinically noticeable but within single-occasion error—monitor and corroborate.
- CC_90_ ≤ |Δ| < CC_95_: exceeds single-occasion error at 90% (not 95%)—interpret cautiously.
- CC_95_ ≤ |Δ| < RCI: exceeds single-occasion error at 95% but not reliably beyond two-occasion error—track trajectory.
- |Δ| ≥ RCI with |Δ| < MID: statistically reliable (95%) yet below clinical importance—consider incremental adjustment.
- |Δ| ≥ max (RCI, MID): statistically reliable (95%) and clinically meaningful; report direction (Δ > 0 improvement; Δ < 0 worsening).

Apply these rules to each scale using the corresponding thresholds in **Table 1**. Set the sign convention (improvement vs. worsening) per scale orientation; reverse if higher scores indicate worse status.

## 4 Discussion

The present study provided an initial validation of the Bipolar Disorder Medication Adherence Battery (BD-MAB), a comprehensive, theory-driven assessment tool designed within the IBM. Across analyses, the BD-MAB showed theory-consistent evidence of validity and internal-consistency reliability, capturing both determinants and outcomes of medication adherence in individuals with BD.

Overall, internal consistency was solid for multi-item BD-MAB scales, with average interitem correlations (AIICs) within the recommended.15–.50 band (41, 57). Self-efficacy showed the tightest coherence (highest AIIC), and attitude and perceived control were in the good–excellent range. The four subjective-norm facets were adequate, consistent with genuine variability in social influences. As expected for a very brief scale capturing contextual breadth, salience of behavior showed modest α but an AIIC at the lower bound of acceptability, supporting its retention. Knowledge & skills demonstrated acceptable homogeneity for a factual content scale. Single-item indicators (habit, intention, behavior) are not evaluated with α; for brief scales, AIIC provides the more informative index (56, 57).

Correlational findings supported key IBM propositions (29) Intention correlated strongly with self-efficacy and attitudes, and the adherence behavior item correlated very strongly with intention and strongly with self-efficacy and attitudes, indicating convergent validity for proximal determinants. Oppositional and pressuring norms showed very weak negative associations with intention, and access showed near-zero relations with most constructs, consistent with its separable, contextual role in the IBM. At the same time, the very strong association between intention and self-efficacy indicates substantial shared variance. From a clinical lens, this highlights that interventions strengthening self-efficacy—such as guided problem-solving, motivational interviewing, or behavioral rehearsal—could enhance adherence intentions and behaviors (59).

External (criterion-related) validity was reinforced by cross-method convergence. The BD-MAB adherence behavior item correlated in the expected direction with serum drug levels, and serum-based adherence status aligned with the BD-MAB dichotomy. When classifying BD-MAB behavior scores of 4–5 as “adherent,” sensitivity was high whereas specificity was comparatively lower, yielding good overall accuracy. Interpreted as screening evidence, the profile suggests the BD-MAB behavior item detects adherent cases well but has more limited precision for identifying non-adherence when used alone. This pattern, i.e., excellent detection of adherent cases with comparatively lower specificity for non-adherence, is typical for self-report adherence indicators and underscores the value of integrating subjective (intention, beliefs, routines) with objective indices (60). Clinicians may therefore use the BD-MAB as a screening tool for probable adherence and as a diagnostic formulation tool to explore modifiable drivers of nonadherence when serum levels or clinical impressions raise concern.

From a clinical standpoint, the BD-MAB’s multidimensional design supports mechanism-specific formulation. The inclusion of habit, barriers, and access helps clinicians identify concrete targets (e.g., routine building, barrier reduction, logistical facilitation) rather than treating “non-adherence” as a unitary construct. This enables individualized care plans that map specific adherence obstacles to targeted psychotherapeutic or case management interventions, such as pillbox training, transportation assistance, or cognitive restructuring of maladaptive beliefs. By operationalizing the IBM’s full structure, the battery aligns with measurement-based care and makes it feasible to monitor hypothesized mechanisms of change during treatment. Furthermore, the battery’s modular design means components can be selectively administered in follow-up visits based on prior results or clinical focus.

Multiple limitations temper interpretation. The cross-sectional design of the present study did not allow evaluation of the BD-MAB’s test–retest reliability or longitudinal sensitivity to change. Similarly, this dataset cannot provide evidence of predictive validity regarding the extent to which BD-MAB subscale scores or total/composite scores predict future clinical outcomes (e.g., relapse, hospitalization, or functional impairment). In this context, although the reported change thresholds (e.g., MID, RCI, and critical change values) offer preliminary guidance for clinical follow-up, these thresholds require anchor-based validation tied to clinically meaningful outcomes. While criterion-related validity was examined via concurrent indicators such as serum drug levels, the prospective capacity of BD-MAB scores to predict relapse, hospitalization, and functional impairment could not be evaluated. Accordingly, repeated assessments are needed to establish test–retest reliability and to validate anchor-based thresholds against clinically meaningful outcomes, which would further clarify the battery’s predictive validity and clinical utility. Although the IBM structure was examined using SEM on scale scores, the item-level factorial structure of the 91-item BD-MAB was not confirmed via CFA in the present sample. Given the length of the item pool and the present sample size, item-level CFA would require larger samples and, ideally, independent replication samples to more robustly evaluate the factorial structure. In addition, classification against the serum-based criterion showed comparatively low specificity, indicating limited precision for detecting non-adherence when the behavior item is used as a stand-alone classifier. Finally, the euthymic, predominantly outpatient BD-I sample may have introduced range restriction, which can attenuate some correlations and limits generalizability to patients in acute depressive, manic, or mixed episodes. Because medication non-adherence is often triggered by symptomatic shifts (e.g., stopping medication during hypomania/mania), the present findings may be conservative relative to samples that include acute phases.

Future research should (a) confirm the BD-MAB’s factorial structure with CFA/SEM, evaluate invariance across key groups, and consider bifactor or higher-order models to parse overlap among proximal determinants; (b) use longitudinal designs to establish test–retest stability, sensitivity to change, and predictive validity for relapse, hospitalization, and functioning; (c) refine lower-α domains and consider expanding single-item constructs; (d) strengthen criterion validity by adding electronic adherence monitoring and pharmacy refill data, enabling ROC/AUC analyses and empirically derived cut points; and (e) derive anchor-based MIDs and decision thresholds tied to meaningful clinical change.

In conclusion, the BD-MAB shows satisfactory internal consistency and theory-consistent concurrent relations with self-report and serum-derived indicators. Current data support its use as a structured, mechanism-focused assessment in individuals with BD—especially for identifying cognitive and contextual barriers to adherence—while underscoring the need for longitudinal and confirmatory work to solidify change thresholds and structural distinctiveness among closely related determinants. Its integration into psychiatric practice may not only clarify the psychological “why” behind nonadherence but also foster more effective, individualized interventions that improve pharmacologic outcomes and reduce relapse risk.

## Data Availability

The raw data supporting the conclusions of this article will be made available by the authors, without undue reservation.
