# Supplementary material for: The Bipolar Disorder Medication Adherence Battery: validity, reliability, and clinical benchmarks
Source: Front Psychiatry. 2026 Feb 19;16:1731246. doi: 10.3389/fpsyt.2025.1731246 (PMC12960553; doi:10.3389/fpsyt.2025.1731246)
Supplement: Supplementary file 1 [file SupplementaryFile1.docx]

### **Supplementary Table S1.** IBM constructs and source instruments used to build the BD-MAB item pool

| IBM construct | Source instruments (item-pool origins) |
| --- | --- |
| Attitude | *Beliefs About Medicines Questionnaire* (Horne, Weinman, & Hankins, 1999); *Drug Attitude Inventory* (Hogan et al., 1983); *Lithium Attitudes Questionnaire* (Harvey, 1991); *Mood Stabilizer Compliance Questionnaire* (Kessing, Hansen, & Bech, 2006); *Self-Report Attitude Toward Psychotropic Medications Questionnaire* (Grover, Chakrabarti, Sharma, & Tyagi, 2014); *Treatment Attitudes Questionnaire* (Johnson & Fulford, 2008). |
| Subjective norms | Authors-generated items (constructed for this study to cover supportive, oppositional, pressuring, and descriptive norms across key referents). |
| Perceived control | *Medication Adherence Self-Efficacy Scale* (Ogedegbe et al., 2003). |
| Self-efficacy | *Medication Adherence Self-Efficacy Scale* (Ogedegbe et al., 2003). |
| Knowledge & skills | *Lithium Attitudes Questionnaire* (Harvey, 1991); *Mood Stabilizer Compliance Questionnaire* (Kessing et al., 2006); *Self-Report Attitude Toward Psychotropic Medications Questionnaire* (Grover et al., 2014). |
| Salience of behavior | *Adherence Starts with Knowledge-20* (Hahn et al., 2008); *Brief Evaluation of Medication Influences and Beliefs* (Dolder et al., 2004); *Lithium Attitudes Questionnaire* (Harvey, 1991); *Self-Report Attitude Toward Psychotropic Medications Questionnaire* (Grover et al., 2014). |
| Environmental constraints | *Lithium Attitudes Questionnaire* (Harvey, 1991). |
| Habit | Authors-generated single item. |
| Intention | Authors-generated single item. |
| Behavior | Authors-generated single item. |

###

### *Note.* IBM = integrated behavioral model.

### **References**

### Dolder, C. R., Lacro, J. P., Warren, K. A., Golshan, S., Perkins, D. O., & Jeste, D. V. (2004). Brief evaluation of medication influences and beliefs: development and testing of a brief scale for medication adherence. Journal of Clinical Psychopharmacology, 24(4), 404-409. <https://doi.org/10.1097/01.jcp.0000130554.63254.3a>

Grover, S., Chakrabarti, S., Sharma, A., & Tyagi, S. (2014). Attitudes toward psychotropic medications among patients with chronic psychiatric disorders and their family caregivers. *Journal of Neurosciences in Rural Practice*, *5*(4), 374-383. <https://doi.org/10.4103/0976-3147.139989>

Harvey, N. S. (1991). The development and descriptive use of the Lithium Attitudes Questionnaire. *Journal of Affective Disorders*, *22*(4), 211-219. <https://doi.org/10.1016/0165-0327(91)90067-3>

Hogan, T. P., Awad, A. G., & Eastwood, R. (1983). A self-report scale predictive of drug compliance in schizophrenics: reliability and discriminative validity. *Psychological Medicine*, *13*(1), 177-183. <https://doi.org/10.1017/S0033291700050182>

Horne, R., & Weinman, J. (1999). Patients’ beliefs about prescribed medicines and their role in adherence to treatment in chronic physical illness. *Journal of Psychosomatic Research*, *47*(6), 555-567. <https://doi.org/10.1016/S0022-3999(99)00057-4>

Johnson, S. L., & Fulford, D. (2008). Development of the treatment attitudes questionnaire in bipolar disorder. *Journal of Clinical Psychology*, *64*(4), 466-481. <https://doi.org/10.1002/jclp.20465>

Kessing, L. V., Hansen, H. V., & Bech, P. (2006). Attitudes and beliefs among patients treated with mood stabilizers. *Clinical Practice and Epidemiology in Mental Health*, *2*, 8. <https://doi.org/10.1186/1745-0179-2-8>

Ogedegbe, G., Mancuso, C. A., Allegrante, J. P., & Charlson, M. E. (2003). Development and evaluation of a medication adherence self-efficacy scale in hypertensive African-American patients. *Journal of Clinical Epidemiology*, *56*(6), 520-529. <https://doi.org/10.1016/S0895-4356(03)00053-2>

**Appendix** Full Item Set of the Bipolar Disorder Medication Adherence Battery (BD-MAB)

**The Bipolar Disorder Medication Adherence Battery (BD-MAB)**

*Buket Ünver, Özlem Sertel Berk, Nesrin Karamustafalıoğlu*

This battery aims to identify the factors that may influence your regular medication-taking behavior. According to the World Health Organization, medication adherence refers to the extent to which an individual takes prescribed medication(s) in the correct dose, at the correct times, and for the prescribed duration. However, it is well established that adherence is influenced by multiple psychological and contextual factors.Accordingly, this questionnaire has been organized into 10 sections, each addressing one dimension of medication adherence. Please read the instructions for each section carefully and select the response option that best represents your thoughts.

When answering, please consider your current use of mood-stabilizing medication (e.g., Lithium, Depakine, Lamictal, Tegretol, Depalept, etc.) and how you intend to take your prescribed mood stabilizer(s) in the appropriate dose and timing over the next three months.

Some items may appear repetitive; however, each provides unique information. Please be patient and answer every question carefully and honestly. There are no right or wrong answers—the questionnaire is about your personal beliefs and experiences.

**ATMA**

**Instructions:**
Below are several statements regarding medication use. Please indicate the degree to which you agree or disagree with each statement. Each statement is rated on a 7-point scale ranging from “Strongly Disagree (1)” to “Strongly Agree (7)”. For each item, circle the number that best reflects your opinion. The higher the number, the more you agree with the statement; the lower the number, the less you agree. Please ensure that you respond to all items and that only one number is circled for each statement.

1. My medications make me feel more normal.
2. My medications help me feel more relaxed.
3. My medications make me feel better.
4. My medications make me feel tired and weak.
5. Having to take medications makes me anxious.
6. The long-term effects of medications sometimes worry me.
7. Becoming too dependent on medications worries me.
8. Even if my medications make me feel better, I worry about possible side effects.
9. I do not like the way medications change my mood.
10. Medications make my thoughts clearer.
11. Despite their side effects, medications are worth taking.
12. Medications protect me from getting worse.
13. Medications make me stronger.
14. Medications help me cope with my problems more effectively.
15. Medications eliminate my depressive and/or manic episodes.
16. Medications can prevent relapses of the illness (episodes).
17. Medications are a better option than seeing faith healers.
18. Medications cause unpleasant side effects.
19. Medications do more harm than good.
20. The side effects of medications are unbearable.
21. Medications dull my personality.
22. Medications take away my cheerful side.
23. Medications may rarely cause permanent harm or damage.
24. Medications worsen the illness in the long run.
25. Medications make people weak and powerless.
26. Medications are addictive.

**NBAMA-S**

**Instructions:**
Below are several statements about how much support you receive from other people regarding your medication-taking behavior.Please indicate the degree to which you agree or disagree with each statement. Each statement is rated on a 7-point scale ranging from “Strongly Disagree (1)” to “Strongly Agree (7)”.For each item, circle the number that best represents your opinion. The more you agree with a statement, the higher the number you should circle. The less you agree, the lower the number you should circle. Please make sure that you respond to all items and that you circle only one number for each statement.

1. My family supports my taking medication.
2. My close friends support my taking medication.
3. Most of the people who are important to me support my taking medication.
4. My psychiatrist supports my taking medication.
5. My relatives support my taking medication.
6. My neighbors support my taking medication.

**NBAMA-O**

**Instructions:**
Below are several statements about how much other people oppose your medication-taking behavior. Please indicate the degree to which you agree or disagree with each statement.Each statement is rated on a 7-point scale ranging from “Strongly Disagree (1)” to “Strongly Agree (7)”. For each item, circle the number that best represents your opinion. The more you agree with a statement, the higher the number you should circle.The less you agree, the lower the number you should circle. Please make sure that you respond to all items and that you circle only one number for each statement.

1. My family opposes my taking medication.
2. My close friends oppose my taking medication.
3. Most of the people who are important to me oppose my taking medication.
4. My psychiatrist opposes my taking medication.
5. My relatives oppose my taking medication.
6. My neighbors oppose my taking medication.

**NBAMA-P**

**Instructions:**
Below are several statements about how much pressure you feel from other people regarding your medication-taking behavior. Please indicate the degree to which you agree or disagree with each statement. Each statement is rated on a 7-point scale ranging from “Strongly Disagree (1)” to “Strongly Agree (7)”. For each item, circle the number that best represents your opinion. The more you agree with a statement, the higher the number you should circle. The less you agree, the lower the number you should circle.Please make sure that you respond to all items and that you circle only one number for each statement.

1. My family pressures me to take my medication.
2. My close friends pressure me to take my medication.
3. Most of the people who are important to me pressure me to take my medication.
4. My psychiatrist pressures me to take my medication.
5. My relatives pressure me to take my medication.
6. My neighbors pressure me to take my medication.

**NBAMA-D**

**Instructions:**
Below are several statements about how different groups or individuals take their prescribed medications at the appropriate doses and times. Please indicate the degree to which you agree or disagree with each statement. Each statement is rated on a 7-point scale ranging from “Strongly Disagree (1)” to “Strongly Agree (7)”. For each item, circle the number that best represents your opinion.The more you agree with a statement, the higher the number you should circle.
Please make sure that you respond to all items and that you circle only one number for each statement.

1. Most people take their prescribed medications at the appropriate doses and times.
2. Many people with illnesses similar to mine take their prescribed medications at the appropriate doses and times.
3. Other members of my family (mother, father, siblings) take their prescribed medications at the appropriate doses and times.
4. My close friends take their prescribed medications at the appropriate doses and times.
5. Most of the people who are important to me take their prescribed medications at the appropriate doses and times.
6. My relatives take their prescribed medications at the appropriate doses and times.
7. My neighbors take their prescribed medications at the appropriate doses and times.

**PCRMA**

**Instructions:**
Below are several statements about environmental factors that may make it easier or harder for you to take your medication as prescribed. Each statement is rated on a 7-point scale ranging from “Strongly Disagree (1)” to “Strongly Agree (7)”.For each item, circle the number that best represents your opinion. The more you agree with a statement, the higher the number you should circle. Please make sure that you respond to all items and that you circle only one number for each statement.

1. It is quite easy to take my medication as part of my daily routine.
2. It is very difficult to keep track of whether I have taken my medication or not.
3. It is very difficult to take my medication while traveling.
4. It is very difficult to take my medication when I am in social settings outside the home (e.g., visiting others).
5. It is very difficult to remember to take my medication.
6. If I have trouble swallowing, it becomes very difficult for me to take my medication.
7. If I need to take other medications as well (e.g., Depakine, lithium), it becomes very difficult for me to take these medications.
8. Taking multiple medications is difficult.
9. Taking my medication feels burdensome.

**SERMA**

**Instructions:**
There may be certain situations in your life that make it more difficult for you to take your medication as prescribed. Below are several statements about how confident you feel in your ability to take your medication despite these difficulties. Each statement is rated on a 7-point scale ranging from “Strongly Disagree (1)” to “Strongly Agree (7)”. For each item, circle the number that best represents your opinion. The more you agree with a statement, the higher the number you should circle. The less you agree, the lower the number you should circle. Please make sure that you respond to all items and that you circle only one number for each statement.

1. I can make taking my medication a part of my daily routine.
2. I can keep track of whether or not I have taken my medication.
3. Even if I experience side effects, I can still manage to take my medication regularly.
4. Even if my family constantly interferes, I can still take my medication.
5. I can request a new prescription when my medication is about to run out.
6. I can take my medication even when I am traveling.
7. I can take my medication even when I am in social settings outside the home.
8. Even if I have difficulty swallowing, I can still take my medication.
9. Even if I have to take other medications as well (e.g., lithium, Depakine), I can still take mine.
10. Even if I need to take multiple medications, I can still take my medication.
11. Even if it is very inconvenient, I can still have my medication prescribed.
12. Even if people who are important to me do not take their medications as prescribed, I can still take mine.

**KAMA**

**Instructions:**
Below are several statements about medication use. Please read each statement carefully and select the option that best describes your answer: TRUE (1) / FALSE (2) / I DON’T KNOW (3).

1. What I know about medications and their effects is correct.
2. On days when you feel better, you can take fewer pills than the prescribed dose.
3. If you forget to take your medication one day, it is better to take an extra dose the next day.
4. The benefits of medication to the body decrease over time with continued use.
5. On days when you feel more depressed, you can take more pills than the prescribed dose.
6. To prevent the medication effect from diminishing, it can be taken every other day instead of daily.
7. It is always better to take your medication at a lower dose than prescribed.

**ECMA**

**Instructions:**
Below are several statements about possible factors that might prevent you from taking your prescribed medications at the appropriate doses and times over the next three months. Each statement is rated on a 7-point scale ranging from “Strongly Disagree (1)” to “Strongly Agree (7)”. The more you agree with a statement, the higher the number you should circle. Please make sure that you respond to all items and that you circle only one number for each statement.

1. Any change in my daily routine prevents me from taking my medication.
2. Frequent changes in the doctors who examine me prevent me from taking my medication.
3. Complicated instructions for medication use prevent me from taking my medication.
4. Doctors not having enough time prevents me from taking my medication.
5. Being unable to ask my doctors the questions I want prevents me from taking my medication.

**SMA**

**Instructions:**
Below are several statements related to accessing your mood-stabilizing medication. Each statement is rated on a 7-point scale ranging from “Strongly Disagree (1)” to “Strongly Agree (7)”. For each item, circle the number that best represents your opinion. The more you agree with a statement, the higher the number you should circle. Please make sure that you respond to all items and that you circle only one number for each statement.

1. I can easily obtain my medication through my health insurance.
2. I have access to a phone to make an appointment.
3. I have access to the internet to make an appointment.
4. I have relatives or close others who can accompany me to the hospital if needed.

**MH**

**Instructions:**

Please indicate how much you agree with the following statement. The statement is rated on a 7-point scale ranging from “Strongly Disagree (1)” to “Strongly Agree (7)”. For each item, circle the number that best represents your opinion.

1. I have managed to take my medication as prescribed by my doctor up to this day.

**IMA**

**Instructions:**

Please indicate how much you agree with the following statement. The statement is rated on a 7-point scale ranging from “Strongly Disagree (1)” to “Strongly Agree (7)”. For each item, circle the number that best represents your opinion.

1. In the next three months, the likelihood that I will take my medication as prescribed is high

**MAB**

**Instructions:**

Please indicate how much you agree with the following statement. The statement is rated on a 5-point scale ranging from “0% (None of the time)” to “100% (All of the time)”. For each item, circle the number that best represents your opinion.

1. How much of your prescribed medication did you take during the past week?
